# Spatial perspective-taking: insights from sensory impairments

**DOI:** 10.1007/s00221-021-06221-6

**Published:** 2021-10-29

**Authors:** Xavier E. Job, Louise P. Kirsch, Malika Auvray

**Affiliations:** 1 Department of Neuroscience, Karolinska Institutet, Solnavägen 9, 17165 Stockholm, Sweden; 2 Institut des Systèmes Intelligents et de Robotique (ISIR), Sorbonne Université, Paris, France; 3 Integrative Neuroscience and Cognition Center (INCC), Université de Paris, Paris, France

**Keywords:** Perspective-taking, Sensory impairments, Perceived self, Spatial perception, Plasticity

## Abstract

Information can be perceived from a multiplicity of spatial perspectives, which is central to effectively understanding and interacting with our environment and other people. Sensory impairments such as blindness are known to impact spatial representations and perspective-taking is often thought of as a *visual* process. However, disturbed functioning of other sensory systems (e.g., vestibular, proprioceptive and auditory) can also influence spatial perspective-taking. These lines of research remain largely separate, yet together they may shed new light on the role that each sensory modality plays in this core cognitive ability. The findings to date reveal that spatial cognitive processes may be differently affected by various types of sensory loss. The visual system may be crucial for the development of efficient allocentric (object-to-object) representation; however, the role of vision in adopting another’s spatial perspective remains unclear. On the other hand, the vestibular and the proprioceptive systems likely play an important role in anchoring the perceived self to the physical body, thus facilitating imagined self-rotations required to adopt another’s spatial perspective. Findings regarding the influence of disturbed auditory functioning on perspective-taking are so far inconclusive and thus await further data. This review highlights that spatial perspective-taking is a highly plastic cognitive ability, as the brain is often able to compensate in the face of different sensory loss.

## Introduction

**Fig. 1:**
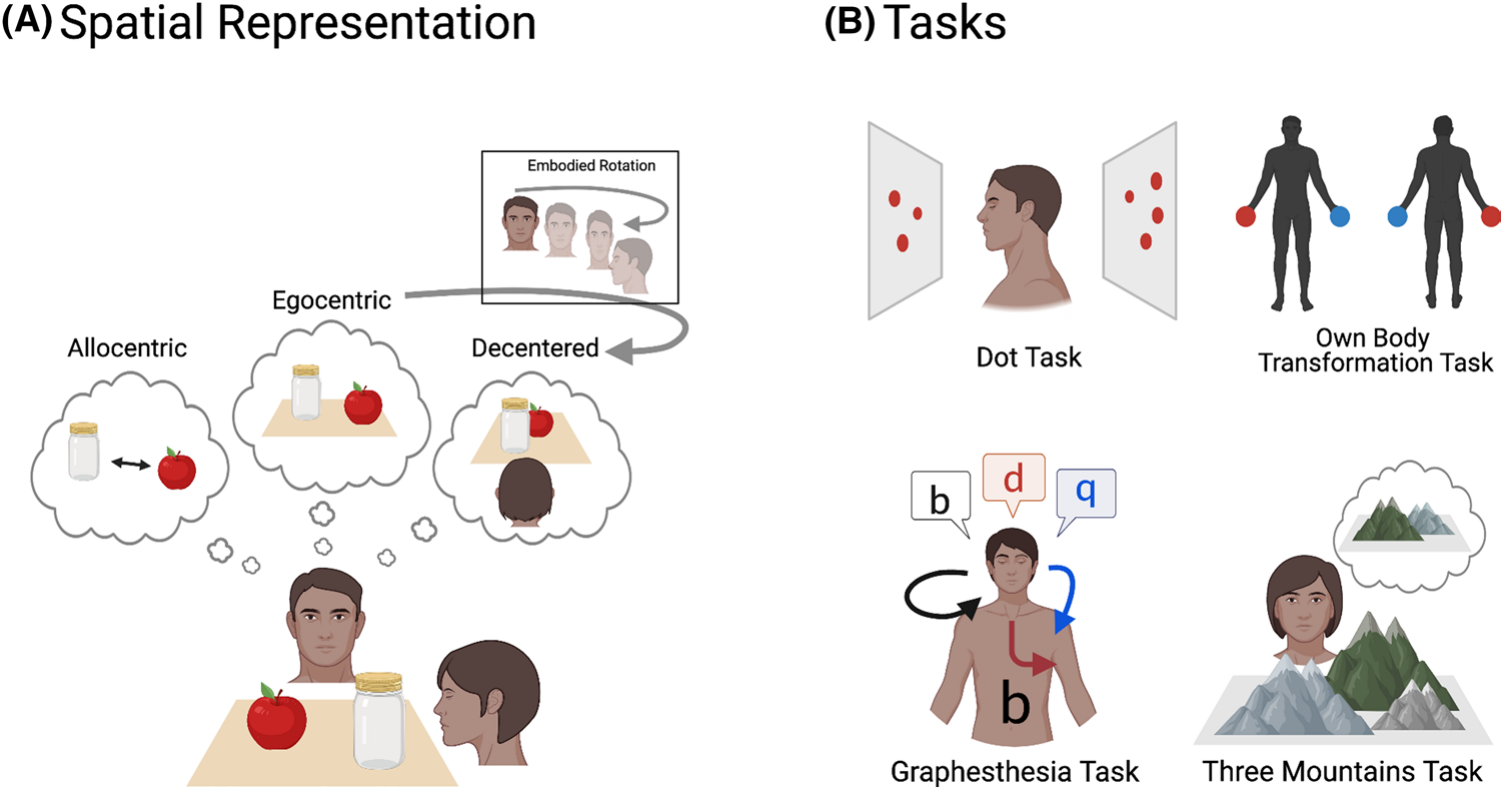
**A** Schematic illustration of an allocentric, egocentric and decentred spatial representation. Note that these representations can also be referred to, respectively, as environment-centred, 1st person/self-centred and 3rd person/altercentric perspective. **B** Schematic illustrations of the behavioural tasks typically used to assess egocentric/decentred spatial perspective-taking. In the Dot Task, participants must count the number of dots that an avatar can or cannot see. In own body transformation tasks, participants make speeded left/right judgements about an avatar that either shares the participant’s posture or not. In the Three Mountains task, participants must accurately judge what a scene looks like from another person’s spatial perspective. In the Graphesthesia Task, ambiguous letter stimuli are traced on the participant’s body and their responses indicate whether they took an egocentric (e.g., trunk-centered or head-centered) or decentred perspective. Figure created with BioRender.com

The ability to adopt a spatial perspective other than one’s own is central to effectively understand and interact with our environment and other people. Objects may be subjectively experienced from the perspective of the self. An *egocentric* perspective can be anchored to the location of the body as a whole, or to body parts such as the head, trunk or even an effector used to perform an action on an object. Objects may also be subjectively experienced from a perspective external to the self, either from another person’s location or merely from any location other than the self. Changing between different perspectives entails a transformation of spatial coordinates. An important distinction is between *allocentric* spatial reference frames and *decentred* spatial perspective-taking (Tversky and Hard 2009). While allocentric representation refers to the construction of cognitive maps that represent the environment independently from the individual, decentering refers to the ability to adopt a perspective anchored to a location outside of one’s body (see Fig. 1A). Thus, allocentric representations concern object-to-object spatial relations typically used during navigation, while decentered representation concerns the ability to adopt another spatial perspective.

How a loss of input in one sensory modality affects task performance in another sensory modality can reveal a great deal about the functional architecture of sensory systems (Pavani and Röder 2012). First, sensory loss may result in impaired performance also in the spared modalities, reflecting a generalised perceptual deficiency. This may indicate that task performance relies on multiple senses working in unison to complete a given cognitive function. Second, performance in the spared modalities may be wholly unaffected by sensory loss, suggesting sensory independence for a given cognitive function. A third eventuality is where the spared sensory modalities are able to compensate, resulting eventually in unimpaired task performance, or in rare cases they ‘hyper-compensate’ resulting in improved performance relative to non-sensory impaired groups. Compensation indicates substantial plasticity of sensory and/or higher order cognitive systems that underlie a given process (Striem-Amit et al. 2012).

The influence of vision loss on the ability to perform spatial tasks has been a longstanding topic of interest, with a focus on how the blind form allocentric spatial representations. Some recent findings suggest that vision loss may also affect spatial perspective taking (SPT) abilities. Separate lines of research have investigated SPT in cases where one sensory modality is disrupted or lost. For example, disruptions to the visual system (such as blindness or visual deprivation), the vestibular system (e.g., vestibular disorders or artificial/natural vestibular stimulation), the proprioceptive system (deafferented patients with somatosensory loss), and the auditory system (deafness) can impact the ability to adopt different spatial perspectives. A clear understanding of how sensory loss affects SPT has been lacking due to these literatures remaining largely separate. Furthermore, multiple different tasks have been used to assess various aspects of perspective-taking. In this review, we bring together these lines of research to shed new light on the impact of sensory loss on the ability to take different spatial perspectives, highlighting the role of each sensory modality in SPT. A related topic is the *remapping* of information from modality specific reference frames (e.g., retinotopic for vision, somatotopic for touch) into an external (i.e., spatiotopic) reference frame (Yamamoto and Kitazawa 2001); however, here we will focus on SPT (egocentred, decentered and allocentric spatial perspectives).

## Vision and spatial perspective-taking

How a loss of vision influences the processing of information across spatial perspectives has been subject to much debate. Broadly, studies have shown that blind individuals rely more on information from egocentric rather than external spatial coordinates (for reviews see Cattaneo et al. 2008; Thinus-Blanc and Gaunet 1997). Some have argued that this reflects a sensitive period in visual development that shapes spatial cognition (Kitchin et al. 1997; Pasqualotto and Newell 2007). Supporting evidence has shown that spatial memory in those without visual experience is often less efficient than those with visual experience for allocentric spatial relations, while performance remains equivalent for egocentric spatial relations (Coluccia et al. 2009; Iachini et al. 2014; Pasqualotto and Newell 2007; Pasqualotto et al. 2013). For example, studies assessing memory for arrays of objects explored through touch reported that individuals with developmental visual experience (blindfolded-sighted and late blind) preferentially represented object locations in an allocentric manner, while those without visual experience (congenitally blind) instead preferentially represented object locations in an egocentric manner (Iachini et al. 2014; Pasqualotto et al. 2013). Thus, allocentric (object-to-object) processing seems dependent on current or past access to visual information (see Arnold et al. 2017b, for a review).

Some have argued that spatial processing in the absence of visual experience is often ameliorated by the use of alternative strategies that instead rely on verbal/semantic, haptic or other non-visual spatial content (Cattaneo et al. 2008). For example, colour is represented in a categorical manner in sighted individuals and this may also be the case for the blind, yet the categorical nature of the representation in the blind may rely instead on abstract knowledge rather than visual experience (Cattaneo et al. 2008). The use of alternative strategies for spatial representation may result in similar or even superior performance for congenitally blind compared to blindfolded-sighted individuals in tasks involving navigation (Passini et al. 1988; Tinti et al. 2006). More recent evidence suggests that the blind may rely less on mental imagery and more on a strategy of verbal rehearsal to complete these types of navigation tasks (Schmidt et al. 2013). Less is known about possible alternative strategies used by the blind to complete spatial perspective taking tasks, yet congenitally blind individuals have also been shown to successfully complete classical Piagetian style (‘three-mountains’) SPT tasks (Heller and Kennedy 1990), in which they have to infer what a person would see from another location on the scene (see Fig. 1B). The blind can also spontaneously adopt a spatial perspective not anchored to the position of their body (i.e., a ‘decentered’ perspective). Spontaneous SPT was observed in blind individuals completing a tactile spatial memory task (Tinti et al. 2018). In this study, participants with little to no visual experience (early blind) and blindfolded-sighted participants explored a 3D tactile map and memorized the location of different landmarks. After the presentation of auditory stimuli from three landmarks positioned on the right, on the left, and in front, participants had to indicate the reciprocal position of the two lateral landmarks. Up to 67% of the blindfolded-sighted group responded from the perspective of the sound source rather than their own perspective. However, up to 53% of the early-blind individuals also responded from the perspective of the sound source. The spontaneous adoption of decentered spatial perspectives was also assessed in a recent study from our group (Job et al. 2021). Participants with varying degrees of visual experience (early blind, late blind, blindfolded-sighted and sighted) completed the Graphesthesia task (Arnold et al. 2016, 2017), a tactile recognition task of ambiguous letter stimuli (b, d, p, and q) presented on the body, for which three perspectives can be adopted (trunk-centred, head-centred and decentered). Even though some of the early and late blind participants spontaneously adopted a decentered perspective, in line with Tinti et al. (2018) and Shimojo et al. (1989), the decentered perspective was adopted significantly more by the blindfolded-sighted group (32% for blindfolded, 20% for sighted and only 6.5% for early and late blind). This suggests that blindness reduces the adoption of decentered perspectives. Moreover, the results showed that both a temporary and permanent lack of vision promotes spontaneous adoption of an egocentred perspective, anchored to the head (> 60% for early and late blind, compared to 30% in sighted). Furthermore, this study investigated not only the influence of vision on the perspective that is adopted spontaneously, but also on the ability to switch between perspectives in the tactile domain. Results showed that the early blind exhibited a greater cost of switching perspectives compared to the sighted, suggesting that early visual experience is important for flexible SPT. This highlights an important ability that is often overlooked, which is the effect of sensory loss on the ability to switch between perspectives. Future studies should explore spatial perspective taking flexibility more systematically.

Inconsistencies in the literature are in part due to differences in experimental tasks as well as the often small and heterogeneous samples tested. As described in the introduction and Fig. 1, one important distinction is between tasks assessing allocentric spatial inferences (*object-object relations*) and tasks assessing the ability to adopt a *decentered* perspective external to one’s own body (Tversky and Hard 2009). Only the latter is thought to involve a transformation of the spatial coordinates of one’s own body. Some authors have highlighted that the ability of the visual system to convey information in parallel might play an important role in processing spatial information (Pasqualotto and Proulx 2012). The unique capability of the visual system to convey information in parallel may not be fully compensated for by the spared modalities. According to this view, a lack of visual experience would result in impaired performance on tasks requiring the parallel representation of allocentric spatial relations (e.g., representing multiple objects relative to each other), while performance would be spared on tasks requiring the serial representation of information from an egocentric perspective (e.g., representing the spatial relationships of individual objects relative to oneself). This could account for some of the contrasting findings, given that visual experience appears to be important for the development of allocentric spatial representation (Iachini et al. 2014; Pasqualotto et al. 2013), yet congenitally blind individuals have been found to spontaneously adopt decentered spatial perspectives (Shimojo et al. 1989; Tinti et al. 2018). Thus, the parallel processing of information afforded by vision may be more crucial for allocentric (object-to-object) spatial relations rather than the ability to decenter one’s spatial perspective. Although studies point toward greater adoption of egocentric perspectives in cases of visual loss, further studies are needed to provide convincing evidence that visual experience is crucial for adopting a decentered spatial perspective.

## The vestibular system and spatial perspective-taking

The vestibular system has classically been understood as serving the control of basic orienting behaviours such as reflexive eye-movements and postural control. Vestibular signals code rotational and linear accelerations of the head during actual own body rotations and are, therefore, thought to be recruited “offline” in the mental simulations of own body rotations (Falconer and Mast 2012). Moreover, in the past 20 years, research has shown a widespread vestibular network, going beyond the low-level reflex motor circuits, including some projections to crucial cortical areas for perspective-taking (such as the parietal cortex; for a review see Ferrè and Haggard 2020). Thus, imagined spatial transformations of one’s own perspective are thought to be mediated by the mental simulation of the mechanisms involved in perceiving actual self-motion, including vestibular processing (Deroualle and Lopez 2014; Palla and Lenggenhager 2014).

### Vestibular disorders

Patients with various vestibular disorders have been found to be slower and less accurate in tasks requiring the mental rotation of their own or another’s body (Candidi et al. 2013; Grabherr et al 2011). More specifically, Candidi et al. (2013) compared patients with unilateral vestibular neuritis, patients with benign unilateral paroxysmal positional vertigo and healthy controls. Their results show that the two groups of patients were less accurate and slower compared to controls both when required to mentally rotate their own body in space (egocentric rotation) and mentally rotate human figures (allocentric rotation) suggesting that unilateral acute disorders of peripheral vestibular input considerably affect the cerebral processes underlying mental rotations. Grabherr et al. (2011) compared patients with unilateral and bilateral vestibular loss with healthy controls and showed that only patients with bilateral vestibular loss have impaired performance in egocentric mental transformation. The disparity between the findings of Candidi et al. (2013) and Grabherr et al. (2011) could result from differences between the individuals included in the unilateral patient groups. The unilateral patient group tested by Candidi et al. were in their acute phase. In this case, the central nervous system receives signals from the inner ear about self-motion and self-orientation that are incongruent with visual and somatosensory signals, thereby creating mismatch between sensory modalities and hence perceptual incoherence. Grabherr et al. (2011) did not test patients in their acute phase, but instead included individuals who underwent labyrinthectomy on average 8 years before testing.

Furthermore, Deroualle et al. (2017) reported no significant differences between patients with bilateral *chronic* vestibular failure and healthy controls for a visual SPT task (the Dot Task) or a tactile SPT task (the Graphesthesia task, Fig. 1B). These patients had functional deficits mild enough not to induce any sensory mismatch or perceptual incoherence. In a following study, Deroualle et al. (2019) tested patients 1 week after a unilateral vestibular neurectomy (deafferentation) and found slowed response times, compared to healthy controls, in a task requiring own body mental rotation, but not for control tasks in which a first-person perspective or 3D object mental imagery were required. Thus, abnormal forms of anchoring the self to the body may arise from perceptual incoherence in *acute* vestibular disorders but not from long-lasting vestibular deafferentation. This suggests that disturbed encoding of own body rotations in the acute phase of a vestibular neurectomy selectively disrupts the mental simulation of own body rotations required for SPT. The effect in Deroualle et al. (2019) was driven by patients who underwent left vestibular neurectomy, as no difference was observed between right vestibular neurectomy patients and healthy controls. The authors reasoned, based on diffusion tensor imaging results of vestibular pathways (Dieterich et al. 2017), that left neurectomy most likely disrupts multisensory processing in bilateral parieto-insular cortex, while right neurectomy likely only disrupts processing in (ipsilateral) right parieto-insular cortex. Thus, it is possible that left vestibular neurectomy evoked deficits in own body mental rotation by disrupting bilateral areas underpinning perspective taking. However, further work is needed to better understand effects of left versus right vestibular deafferentation.

### Vestibular stimulation

Further evidence that the vestibular system plays a role in SPT has used either caloric vestibular stimulation (CVS) or galvanic vestibular stimulation (GVS). Although vestibular stimulation cannot be considered equivalent to vestibular loss*,* several recent findings suggest that perturbing the normal functioning of the vestibular system can affect performance on tasks of SPT. Findings show impaired performance on egocentric mental transformation tasks following right-anodal GVS (Lenggenhager et al. 2008) as well as following binaural-bipolar pseudorandom sum of sines stimulation at suprathreshold (peak amplitude between 3.5 and 5 mA) compared to subthreshold (peak amplitude between 0 and 1 mA; Dilda et al. 2012). More specifically, in Lenggenhager et al.’s (2008) study, participants made left–right judgments about an asymmetrical object (picture of a plant that extended to the left or the right) or a human body with an extended left or right arm. The stimuli were rotated either a small amount (60 degrees) or a large amount (120 degrees) to manipulate the difficulty of the left–right judgement. Afterwards, participants were divided into two groups based on whether they reported using an object-based mental transformation strategy (i.e., “I imagined the picture turning”) or an egocentric mental transformation strategy (i.e., “I imagined myself turning”). The authors found an effect of right-anodal binaural-bipolar GVS for large angles of rotation only for participants who reported using an egocentric mental transformation strategy. No effects of GVS were found for participants who reported using an object-based mental transformation strategy. This suggests that egocentric mental transformation simulates the properties of physical egocentric transformation, since real body movements involve vestibular processes whereas physical object transformation does not. Why only right, but not left, anodal GVS affected the more demanding egocentric transformations (i.e., larger angle of rotation) is not clear. However, right-anodal GVS has been shown to stimulate bilateral vestibular areas, while the effects of left-anodal GVS are confined to the contralateral (right) hemisphere (Fink et al. 2003), so it is possible that bilateral inhibition of vestibular areas is necessary to disrupt egocentric transformations.

Certain types of vestibular stimulation have been found to improve, rather than disrupt, performance on SPT tasks. For example, using caloric vestibular stimulation (CVS) to mimic rightward head rotations, Falconer and Mast (2012) found that stimulation facilitated congruent egocentric mental transformations (to the right), with no effect of stimulation for the mental transformation of hand or letter stimuli. Furthermore, low-intensity GVS has been found to promote the adoption of egocentred perspectives rather than the adoption of another’s perspective. For example, Pavlidou et al. (2018) observed that a 1 mA left-anodal binaural-bipolar square-wave stimulation (GVS) reduced the interference from another’s visual spatial perspective. Low-intensity GVS (binaural-bipolar boxcar pulse of 1 mA) also increased the adoption of a first-person perspective, rather than a third-person perspective, to perceive ambiguous tactile stimuli traced on the forehead (i.e., Graphesthesia task; Ferrè et al. 2014). The authors proposed that low-intensity vestibular stimulation increases the natural tendency of the vestibular system to anchor the self to the physical body, suggesting a vestibular contribution to embodied self-location. Thus, the vestibular system may naturally promote an egocentred spatial perspective.

Three studies have used natural vestibular stimulation with rotating platforms in combination with SPT tasks to probe the contribution of the vestibular system to SPT. In one of these studies, performance on an own body transformation task was found to be disrupted by whole body “Coriolis motion”, known to provoke aberrant stimulation of the vestibular system (Gardner et al. 2017). Coriolis motion is a highly disruptive vestibular stimulus, known to impair cognitive performance and induce motion sickness. On the other hand, two studies (Deroualle et al. 2015; van Elk and Blanke 2014) observed direction specific improvements in visual SPT task performance when the stimulation matched the direction needed to rotate one’s imagined body in line with another’s perspective. These effects were all task-specific, as they did not influence either 3D mental object rotation or control tasks requiring a reconfiguration of spatial mappings from one’s own visual–spatial perspective. Together these studies demonstrate the importance of vestibular signals for efficient visual SPT. While perturbing the vestibular system can disrupt embodied mental transformations, vestibular stimulation can also improve embodied transformations when the stimulation matches the direction of rotation needed to complete the task. In addition, these studies suggest that rotating platforms might provide a means to improve performance in cases of vestibular impairment. However, it is difficult to rule out a contribution of accompanying proprioceptive, somatosensory and visual input during natural vestibular stimulation.

Taken together, results from vestibular disorders and vestibular stimulation studies suggest that the vestibular system is involved in body transformation, but not necessarily object transformation, in both visual and tactile SPT. Overall, the vestibular system plays a role in anchoring the perceived self to the body (either by promoting the egocentric perspective or disrupting the decentered one), a cognitive function that is crucial to imagined self-rotation and thus SPT.

## Proprioception and spatial perspective-taking

Proprioception is the sense of position and movement of body segments, mediated by signals from receptors in the muscles, tendons, joints and skin. This sense can be lost in cases of deafferentation (Cole and Paillard 1995). It is well known that our position in space (i.e., proprioceptive information) influences performance on tasks of spatial perspective-taking. For example, making judgements about whether an object is to the left or the right of someone else are made significantly faster when the perceiver shares the same body posture (Kessler and Rutherford 2010; Kessler and Thomson 2010). Furthermore, incongruent visuo-proprioceptive signals between one's own body posture and someone else's decreases the likelihood of adopting their visuo-spatial perspective (Pavlidou et al. 2019). However, little is known about the influence of proprioceptive loss on SPT. The results of one study with a deafferented patient (GL) suggested that the loss of proprioception causes an impaired egocentric processing (Blouin et al. 1995). Another study (Bringoux et al. 2016) investigated whether a massive loss of somatosensory inputs changes the perception of an external object’s spatial orientation and self-orientation perception. One deafferented patient’s perception of external orientation was found to strikingly depend on visual inputs. However, despite visual and vestibular cues that could be used to compensate for impaired proprioception in a self-orientation task, the patient never perceived being tilted, contrary to healthy controls who were able to detect changes in self-orientation relative to vertical.

Since then, two studies have investigated the impact of proprioceptive loss on navigation and SPT. Renault et al. (2018) compared the ability to form spatial models in two patients chronically deprived of proprioception (GL and IW) and healthy control participants. The participants listened to two types of verbal descriptions of a spatial environment one according to an egocentric (route) reference frame and the other according to an allocentric (survey) reference frame. Performance was assessed by a distance-comparison task in which both accuracy and reaction times were measured. Contrary to the authors’ predictions, proprioceptive loss did not systematically impair accuracy when the spatial environment was described in egocentric coordinates. While one deafferented individual (GL) made more errors than controls, the other (IW) made less errors, both without obvious differences as a function of the reference frame condition. Although GL and IW were able to perform the task, they were slower to respond than controls. This suggests that proprioceptive impairments did not influence the capacity to form accurate spatial representations; however, it can slow down the processing of these spatial representations. This could reflect a deficit in forming or in reporting these spatial representations; however, the study was not able to disentangle the two. Arnold et al. (2019) investigated the role of somatosensory and visual information in adopting self-centered and decentered spatial perspectives in the same deafferented patients (GL and IW) and age matched controls. Participants performed a graphesthesia task, consisting of the recognition of ambiguous letters (b, d, p, and q) traced on the forehead (as described in Part 2. and shown in Fig. 1B). The two patients had intact somatosensory processing on the forehead as they were impaired either from the nose down (for GL) or from the neck down (for IW). While IW mainly adopted a decentered perspective, GL clearly preferred an egocentered one. In contrast to the healthy controls, there was no effect of body posture on the adopted perspective in the deafferented patients. This also did not support the hypothesis that deafferented patients have a deficit in adopting an egocentric perspective. It is difficult to draw any strong conclusions with only two patients tested in the above-mentioned studies and future research should investigate the role of proprioception on SPT further. Given that deafferented patients are extremely rare, future research on healthy populations using alternative paradigms that perturb normal proprioceptive functioning (e.g., virtual reality or illusions) should also be conducted. This could help to corroborate findings that disrupted proprioception is associated with impaired egocentric processing, and that this impairment might be compensated by other sensory inputs.

## Audition and spatial perspective-taking

How auditory loss affects SPT is largely unknown, yet some have suggested that deaf signers may approach SPT tasks differently because of their extensive experience with language in the visual–spatial modality (Secora and Emmory 2019, 2020). Recent studies have investigated whether those with hearing loss approach perspective-taking tasks by engaging more or less in strategies involving embodied self-rotation or rule-based strategies. Secora and Emmory (2019) did not find any significant differences between a group of deaf signers (*n* = 44) and a group of hearing non-signers on a visual–spatial perspective-taking task (“Three Buildings Task”, a version of the classical Three Mountains Task shown in Fig. 1B), in line with earlier studies with deaf children (Peterson and Peterson 1990). However, hearing non-signers with better social abilities, as measured by the Autism-Spectrum Quotient (Baron-Cohen et al. 2001), performed better on the VSPT task, while deaf signers with poorer social abilities performed better on the VSPT task. Therefore, social abilities and VSPT skills relate differently for deaf and hearing individuals, possibly due to differences in communication modality. van Dijk et al. (2013) showed improved haptic orientation processing in deaf individuals, indicating that they could better identify allocentric spatial coordinates. Finally, Zhang et al. (2014) showed that egocentric processing was compromised after early deafness, whereas allocentric processing remained intact. Only the non-deaf group showed *asymmetrical interference*, such that irrelevant egocentric information interfered more with allocentric information than the reverse. The deaf group showed symmetrical interference. Together, these studies show that a loss of hearing could either impair egocentric spatial processing or promote allocentric processing. The mechanisms underlying effects of auditory loss on perspective taking remain to be investigated. Thus, further data are needed to elucidate the role the auditory system plays in SPT.

## Conclusion

**Fig. 2:**
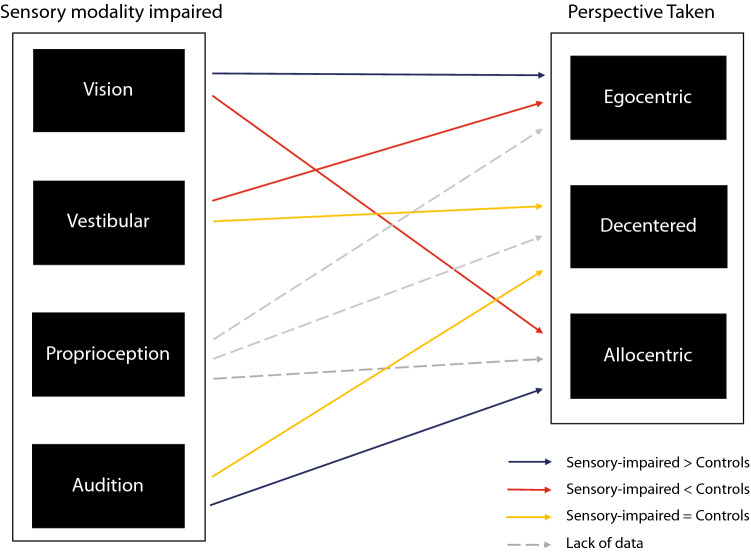
Schema of the impacts of sensory loss on the perspective adopted spontaneously. The solid line indicates a known and non-ambiguous effect. Dark blue lines indicate that sensory impairment in this modality increases the adoption of that perspective, relative to non-sensory impaired controls; the red lines indicate that sensory impairment decreases the ability to adopt that perspective; and the yellow lines indicate no difference found between those with sensory loss and controls. Note that when inconsistencies in results were found, links are not represented (e.g. the effect of vision loss on the ability to take a decentred perspective). Dashed grey lines indicate a lack of data for effects of proprioceptive loss on the perspective adopted (as only two patients were tested)

Research on sensory impairments is advancing our understanding of spatial perspective-taking. In this review, separate research on SPT in cases of sensory loss or deprivation was reviewed. The findings first demonstrate that SPT is affected by visual, vestibular, and proprioceptive impairments (as summarized in Fig. 2 and Table 1). Therefore, rather than individual sensory modalities representing spatial information independently from each other, SPT is most likely a multisensory phenomenon that relies on the senses working in unison.

**Table 1:** A summary of the studies investigating the impact of sensory loss on spatial perspective-taking (SPT)

Authors	Participants (*n*)	Task	Cognitive processes	Findings
Visual impairment				
Shimojo et al. (1989)	Early blind (8) Sighted (8)	Manual graphesthesia task	Tactile SPT	Early blind and sighted have equal performance in decentering
Heller and Kennedy (1990)	Congenitally blind (9) Late blind (9), Blindfolded (9)	Three mountains task	Tactile SPT	Congenitally blind, late blind and blindfolded have equal performance in decentering
Pasqualotto and Newell. (2007)	Congenitally blind (10) Late blinds (12) Sighted (10)	Tactile spatial memory	Allocentric representation	Early blind have lower performance than late blind and controls (allocentric)
Coluccia et al. (2009)	Congenitally blind (16) Sighted (16)	Tactile spatial memory	Allocentric—egocentric representation	Congenitally blind have lower performance than sighted in an allocentric task and equal performance in an egocentric one
Pasqualotto et al. (2013)	Early blind (10) Late blind (10) Blindfolded (10)	Tactile spatial memory	Allocentric—egocentric representation	Blindfolded and late blind preferentially used an allocentric perspective and congenitally blind an egocentric one
Iachini et al. (2014)	Congenitally blind (22) Late blind (22) Blindfolded (44) Sighted (44)	Tactile spatial memory	Allocentric—egocentric representation	Increased difficulty with allocentric representation for larger scale arrays in congenitally blind
Ruggiero et al. (2018)	Congenitally blind (12) Blindfolded (12) Sighted (12)	Tactile spatial memory	Allocentric—egocentric representation	Difficulty for congenitally blind when switching from allocentric to egocentric representations. Deficit in processing allocentric representations in non-switching conditions
Tinti et al. (2018)	Early blind (15) Blindfolded (15)	Tactile spatial memory	Tactile SPT	Congenitally blind spontaneously adopted another’s perspective similarly to blindfolded controls
Job et al. (2021)	Early blind (28) Late blind (32) Blindfolded (38) Sighted (30)	Automatized Graphesthesia task	Tactile SPT + switching	Early, late blinds, and blindfolded spontaneously adopt more often an egocentric perspective. Early and late blinds adopt spontaneously a decentered perspective less often than sighted and blindfolded. Early blind have lower performance than sighted in switching perspectives
Vestibular impairment				
Grabherr et al. (2011)	Bilateral vestibular patients (8) Unilateral vestibular patients (15) Controls (14)	Own body and object mental rotation	Egocentric and object rotation	Participants with bilateral vestibular loss showed impaired performance in egocentric mental transformation. No deficit for unilateral vestibular lesions group, and no differences between right- and left-sided labyrinthectomized patients
Candidi et al. (2013)	Vestibular disorders: VN (9) BPPN (14) Controls (16)	Own body and human figure mental rotation	Egocentric and allocentric rotation	VN and BPPV patients are more impaired than controls in performing mental rotation tasks of both their own body (egocentric) and human figures (allocentric)
Deroualle et al. (2017)	Chronic bilateral vestibular failure (BVF, 23) Controls (23)	Dot task Manual Graphesthesia task	Visual SPT Tactile SPT	No differences between the groups
Deroualle et al. (2019)	Left vestibular neurectomy (12) Right vestibular neurectomy (11) Controls (23)	3D objects mental imagery Virtual ball tossing game	Mental imagery and visual SPT	Left vestibular neurectomy leads to deficits in decentering
Proprioceptive impairment				
Renault et al. (2018)	Deafferented patients (2) Controls (16)	Audio map memory task	Allocentric—egocentric representation	Deafferented patients have higher RTs when performing egocentric and allocentric tasks
Arnold et al. (2019)	Deafferented patients (2) Controls (20)	Automatized Graphesthesia task	Tactile spontaneous SPT	While one patient mainly adopted a decentered perspective the other adopted an egocentric one
Auditory impairment				
Peterson and Peterson (1990)	Deaf children (24) Controls (10)	Three mountains Task (equivalent)	Visual SPT	Deaf children and controls have equal performance
van Dijk et al. (2013)	Deaf signers (15) Hearing signers (16) Hearing non-signers (16)	Haptic parallel setting task	Allocentric representation	Deaf signers have better performance than hearing signers and non-signers
Zhang et al. (2014)	Congenital deaf (17, 18) Controls (20, 20)	Allocentric/egocentric judgment tasks	Allocentric—egocentric representation	No impact of congenital deafness for allocentric processing, but deficit in egocentric processing
Secora and Emmory (2019)	Deaf signers (44) Hearing non-signers (45)	Three buildings Task	Visual SPT	Deaf signers and hearing non-signers have equal performance

However, the findings also reveal that a loss of one sensory modality can modify (either impair or promote) the representation of egocentric, decentered or allocentric information while the loss of another sensory modality can have a different effect. Not all of these eventualities have been adequately tested so far, but the fact that spatial representations can be affected differently depending on which sensory modality is impaired suggests that certain modalities are more important than others for certain spatial representations. The evidence from sensory impairments so far suggests that both the vestibular and proprioceptive systems likely play an important role in anchoring the perceived self to the physical body. This is thought to facilitate imagined self-rotations required to adopt another’s spatial perspective. In addition, the transformation of perspectives from multiple sensory modalities into a unified self-centered perspective, which allows the observer to adopt a unique point of view on the external world, relies on vestibular and somatosensory information (see also Arnold et al. 2017). Visual impairments often decrease performance in tasks requiring allocentric (object-object) spatial representation. Thus, the visual system may be crucial for the development of efficient allocentric representation, perhaps due to its capability for parallel processing of information. However, the role of vision in adopting another’s spatial perspective (i.e., ‘decentering’) is less clear and awaits further data. Regarding auditory loss, although the reviewed studies point toward either an impaired egocentric spatial processing or an increase in allocentric processing, further data are needed to confirm the role of audition in SPT. The studies reviewed here highlight the different roles that each sensory modality might play in the ability to adopt a given perspective. It should; however, be underlined that only one study so far investigated whether sensory loss affects SPT in different modalities (e.g., whether vestibular failure impacts SPT in the tactile and visual domain; Deroualle et al. 2017). Future studies should investigate this further to better understand the contribution and compensation mechanisms that are potentially involved as a function of sensory modality. Moreover, little is known about the potential contribution of different sensory modalities to the flexibility of SPT. Flexibly and appropriately switching between the representation of information in egocentric and decentred perspectives is likely critical to efficient SPT, and future research should investigate this ability.

Proprioceptive and vestibular signals predominantly provide proximal information originating from the body, whereas visual signals mostly provide distal information originating from outside the body. The distinction between *proximal* and *distal* senses (or between inside and outside the body) could be one explanation for the role that different senses play in adopting an egocentric or a decentered perspective; with a weighting of external versus internal information and subtle balance between both perspectives. However, this hypothesis requires testing in future studies.

## References

[CR1] Arnold G, Spence C, Auvray M (2016). Taking someone else’s spatial perspective: natural stance or effortful decentring?. Cognition.

[CR2] Arnold G, Spence C, Auvray M (2017). A unity of the self or a multiplicity of locations? How the graphesthesia task sheds light on the role of spatial perspectives in bodily self-consciousness. Conscious Cogn.

[CR3] Arnold G, Sarlegna FR, Fernandez LG, Auvray M (2019). Somatosensory loss influences the adoption of self-centered versus decentered perspectives. Front Psychol.

[CR347] Baron-Cohen S, Wheelwright S, Skinner R, Martin J, Clubley E (2001) The autism-spectrum quotient (AQ): evidence from asperger syndrome/high-functioning autism, malesand females, scientists and mathematicians. J Autism Dev Disord 31(1):5–17 10.1023/a:1005653411471 11439754

[CR4] Blouin J, Vercher JL, Gauthier GM, Paillard J, Bard C, Lamarre Y (1995). Perception of passive whole-body rotations in the absence of neck and body proprioception. J Neurophysiol.

[CR5] Bringoux L, Scotto Di Cesare C, Borel L, Macaluso T, Sarlegna FR (2016). Do visual and vestibular inputs compensate for somatosensory loss in the perception of spatial orientation? Insights from a deafferented patient. Front Hum Neurosci.

[CR6] Candidi M, Micarelli A, Viziano A, Aglioti SM, MinioPaluello I, Alessandrini M (2013). Impaired mental rotation in benign paroxysmal positional vertigo and acute vestibular neuritis. Front Hum Neurosci.

[CR7] Cattaneo Z, Vecchi T, Cornoldi C, Mammarella I, Bonino D, Ricciardi E, Pietrini P (2008). Imagery and spatial processes in blindness and visual impairment. Neurosci Biobehav Rev.

[CR8] Cole J, Paillard J (1995). Living without touch and peripheral information about body position and movement: studies with deafferented subjects. The body and the self.

[CR9] Coluccia E, Mammarella IC, Cornoldi C (2009). Centred egocentric, decentred egocentric, and allocentric spatial representations in the peripersonal space of congenital total blindness. Perception.

[CR10] Deroualle D, Lopez C (2014). Toward a vestibular contribution to social cognition. Front Integr Neurosci.

[CR11] Deroualle D, Borel L, Devèze A, Lopez C (2015). Changing perspective: the role of vestibular signals. Neuropsychologia.

[CR12] Deroualle D, Toupet M, Van Nechel C, Duquesne U, Hautefort C, Lopez C (2017). Anchoring the self to the body in bilateral vestibular failure. PLoS ONE.

[CR13] Deroualle D, Borel L, Tanguy B, Bernard-Demanze L, Devèze A, Montava M (2019). Unilateral vestibular deafferentation impairs embodied spatial cognition. J Neurol.

[CR14] Dieterich M, Kirsch V, Brandt T (2017). Right-sided dominance of the bilateral vestibular system in the upper brainstem and thalamus. J Neurol.

[CR15] Dilda V, MacDougall HG, Curthoys IS, Moore ST (2012). Effects of galvanic vestibular stimulation on cognitive function. Exp Brain Res.

[CR16] Falconer CJ, Mast FW (2012). Balancing the mind: vestibular induced facilitation of egocentric mental transformations. Exp Psychol.

[CR17] Ferrè ER, Haggard P (2020). Vestibular cognition: state-of-the-art and future directions. Cogn Neuropsychol.

[CR18] Ferrè ER, Lopez C, Haggard P (2014). Anchoring the self to the body: vestibular contribution to the sense of self. Psychol Sci.

[CR19] Fink GR, Marshall JC, Weiss PH, Stephan T, Grefkes C, Shah NJ, Zilles K, Dieterich M (2003). Performing allocentric visuospatial judgments with induced distortion of the egocentric reference frame: an fMRI study with clinical implications. Neuroimage.

[CR20] Gardner MR, Stent C, Mohr C, Golding JF (2017). Embodied perspective-taking indicated by selective disruption from aberrant self motion. Psychol Res.

[CR21] Grabherr L, Cuffel C, Guyot JP, Mast FW (2011). Mental transformation abilities in patients with unilateral and bilateral vestibular loss. Exp Brain Res.

[CR22] Heller MA, Kennedy JM (1990). Perspective taking, pictures, and the blind. Percept Psychophys.

[CR23] Iachini T, Ruggiero G, Ruotolo F (2014). Does blindness affect egocentric and allocentric frames of reference in small and large scale spaces?. Behav Brain Res.

[CR24] Job X, Arnold G, Kirsch L, Auvray M (2021). Vision shapes tactile spatial perspective-taking. J Exp Psychol Gen.

[CR25] Kessler K, Rutherford H (2010). The two forms of visuo-spatial perspective taking are differently embodied and subserve different spatial prepositions. Front Psychol.

[CR26] Kessler K, Thomson LA (2010). The embodied nature of spatial perspective taking: embodied transformation versus sensorimotor interference. Cognition.

[CR27] Kitchin RM, Blades M, Golledge RG (1997). Understanding spatial concepts at the geographic scale without the use of vision. Prog Hum Geogr.

[CR28] Lenggenhager B, Lopez C, Blanke O (2008). Influence of galvanic vestibular stimulation on egocentric and object-based mental transformations. Exp Brain Res.

[CR29] Palla A, Lenggenhager B (2014). Ways to investigate vestibular contributions to cognitive processes. Front Integr Neurosci.

[CR30] Pasqualotto A, Newell FN (2007). The role of visual experience on the representation and updating of novel haptic scenes. Brain Cogn.

[CR31] Pasqualotto A, Proulx MJ (2012). The role of visual experience for the neural basis of spatial cognition. Neurosci Biobehav Rev.

[CR32] Pasqualotto A, Spiller MJ, Jansari AS, Proulx MJ (2013). Visual experience facilitates allocentric spatial representation. Behav Brain Res.

[CR33] Passini R, Delisle J, Langlois C, Proulx G (1988). Wayfinding information for congenitally blind individuals. J vis Impair Blind.

[CR34] Pavani F, Röder B (2012). Crossmodal plasticity as a consequence of sensory loss: insights from blindness and deafness. The new handbook of multisensory processes.

[CR35] Pavlidou A, Ferrè ER, Lopez C (2018). Vestibular stimulation makes people more egocentric. Cortex.

[CR36] Pavlidou A, Gallagher M, Lopez C, Ferrè ER (2019). Let's share our perspectives, but only if our body postures match. Cortex.

[CR37] Peterson CC, Peterson JL (1990). Sociocognitive conflict and spatial perspective-taking in deaf children. J Appl Dev Psychol.

[CR38] Renault AG, Auvray M, Parseihian G, Miall RC, Cole J, Sarlegna FR (2018). Does proprioception influence human spatial cognition? A study on individuals with massive deafferentation. Front Psychol.

[CR346] Ruggiero G, Ruotolo F, Iachini T (2018) Congenital blindness limits allocentric to egocentric switching ability. Exp Brain Res 236(3):813–820 10.1007/s00221-018-5176-8 29340716

[CR39] Schmidt S, Tinti C, Fantino M, Mammarella IC, Cornoldi C (2013). Spatial representations in blind people: the role of strategies and mobility skills. Acta Psychol.

[CR40] Secora K, Emmorey K (2019). Social abilities and visual-spatial perspective-taking skill: deaf signers and hearing nonsigners. J Deaf Stud Deaf Educ.

[CR41] Secora K, Emmorey K (2020). Visual-spatial perspective-taking in spatial scenes and in american sign language. J Deaf Stud Deaf Educ.

[CR42] Shimojo S, Sasaki M, Parsons LM, Torii S (1989). Mirror reversal by blind subjects in cutaneous perception and motor production of letters and numbers. Percept Psychophys.

[CR345] Striem-Amit E, Bubic A, Amedi A (2012) Neurophysiological mechanisms underlying plastic changes and rehabilitation following sensory loss in blindness and deafness. In: The neural bases of multisensory processes 22593863

[CR43] Thinus-Blanc C, Gaunet F (1997). Representation of space in blind persons: vision as a spatial sense?. Psychol Bull.

[CR44] Tinti C, Adenzato M, Tamietto M, Cornoldi C (2006). Visual experience is not necessary for efficient survey spatial cognition: evidence from blindness. Q J Exp Psychol.

[CR45] Tinti C, Chiesa S, Cavaglià R, Dalmasso S, Pia L, Schmidt S (2018). On my right or on your left? Spontaneous spatial perspective taking in blind people. Conscious Cogn.

[CR46] Tversky B, Hard BM (2009). Embodied and disembodied cognition: spatial perspective-taking. Cognition.

[CR47] van Elk M, Blanke O (2014). Imagined own-body transformations during passive self-motion. Psychol Res.

[CR48] van Dijk R, Kappers AM, Postma A (2013). Superior spatial touch: improved haptic orientation processing in deaf individuals. Exp Brain Res.

[CR49] Yamamoto S, Kitazawa S (2001). Reversal of subjective temporal order due to arm crossing. Nat Neurosci.

[CR50] Zhang M, Tan X, Shen L, Wang A, Geng S, Chen Q (2014). Interaction between allocentric and egocentric reference frames in deaf and hearing populations. Neuropsychologia.

